# From Learning to Decision-Making: A Cross-Sectional Survey of a Clinical Pharmacist-Steered Journal Club

**DOI:** 10.3390/pharmacy5010003

**Published:** 2017-01-12

**Authors:** Sherine Ismail, Sara Al Khansa, Mohammed Aseeri, Hani Alhamdan, K. H. Mujtaba Quadri

**Affiliations:** 1 King Abdullah International Medical Research Center, King Saud bin Abdulaziz University for Health Sciences, Pharmaceutical Care Department, King Abdulaziz Medical City, Ministry of National Guard Health Affairs, Jeddah 21423, Saudi Arabia; khansasa@ngha.med.sa (S.A.K.); aseerima@ngha.med.sa (M.A.); hamdanhs@ngha.med.sa (H.A.); 2 National University of Medical Sciences, The Mall, Rawalpindi 44000, Pakistan; deanresearch@numspak.edu.pk

**Keywords:** journal club, learning, decision-making, clinical pharmacist, evidence-based practice

## Abstract

Journal clubs have been traditionally incorporated into academic training programs to enhance competency in the interpretation of literature. We designed a structured journal club (JC) to improve skills in the interpretation of literature; however, we were not aware of how learners (interns, residents, clinical pharmacists, etc.) would perceive it. We aimed to assess the perception of learners at different levels of pharmacy training. A cross-sectional design was used. A self-administered online survey was emailed to JC attendees from 2010–2014 at King Abdulaziz Medical City, Jeddah, Saudi Arabia. The survey questions included: introduction sessions, topic selection, JC layout, interaction with the moderator, and decision-making skills by clinical pharmacists. The response rate was 58/89 (65%); 52/54 (96%) respondents believed that JC adds to their knowledge in interpreting literature. Topic selection met the core curriculum requirements for credentials exams for 16/36 (44.4%), while 16/22 (73%) presenters had good to excellent interaction with the moderator. JC facilitated decision-making for 10/12 (83%) of clinical pharmacists. The results suggest that clinical pharmacist-steered JC may serve as an effective tool to empower learners at different levels of pharmacy practice, with evidence-based principles for interpretation of literature and guide informed decision-making.

## 1. Introduction

Journal clubs have been traditionally incorporated into academic training programs to enhance competency in the interpretation of literature [1]. A systematic review highlighted the potential influence of a journal club to enhance critical thinking and promote knowledge in clinical research, bridging theory to practice and subsequently improving clinical practice [2]. Furthermore, several journal club models conducted over training periods have been reported to promote life-long learning and maintain certifications for residents and faculty [3]. Harris et al. (2011) conducted a systematic review of 18 studies to assess the effectiveness of JCs. Five studies (5/11) reported an improvement in reading behavior, (7/7) showed confidence in critical appraisal, (5/7) presented critical appraisal test scores, and (5/7) demonstrated an ability to implement study findings; however, pooling of the study results was difficult due to heterogeneous interventions and it was not obvious how JCs would guide evidence-based decisions [4]. Additionally, Matthews DC (2011) pointed that Harris et al. (2011) identified two key points: first, the success of the JC is dependent on learner-centered approach; second, there is no standard layout for the journal club, despite identifying the essential elements such as skilled mentorship, structured format for literature review, and the use of active learning techniques [5].

The statement of the American Society of Health-System Pharmacist for formulary management identifies pharmacists as crucial members of multidisciplinary teams to nourish the Pharmacy and Therapeutics Committee (P & T) proceeding with critical evaluation of literature and to guide P & T evidence-based decisions for drug alternatives [6]. Therefore, it is prudent to promote critical thinking and sharpen critical appraisal skills for the interpretation of literature for pharmacy practitioners at different levels of training and expertise, through structured JCs to fulfill leadership obligations and organizational decisions [7].

The structure of an effective JC has been identified in literature with several features such as a trained leader to mentor the discussion and select scientific papers for review, mandatory attendance, regular scheduled meetings, disseminating JC materials, and the use of standard critical appraisal tools [8]. Mcleod RS et al. (2010) published a randomized control trial comparing moderated JCs versus Internet-based JCs for surgical residents and reported higher scores in critical appraisal skills with moderated JCs [9].

We designed a structured JC steered by a clinical pharmacist certified in clinical research to improve skills in interpretation of literature in the pharmaceutical care department at King Abdulaziz Medical City, Ministry of National Guard Health Affairs, Jeddah, Saudi Arabia. However, we were not aware of the perception of learners attending the JC (interns, residents, clinical pharmacists, etc.) on their practice, skills and decision-making process. Therefore, our aim was to assess the perception of learners at different levels of pharmacy training regarding the structure and utility of the JC steered by a clinical pharmacist. Additionally, we evaluated the perception of pharmacists regarding its role in guiding formulary decisions and informing clinical practice.

## 2. Materials and Methods

### 2.1. Development and Validation of the Survey Tool

An online survey was designed through SurveyMonkey by the clinical pharmacist in May 2014 and validated for language, wording, and content through a pilot study on 4 volunteering health care professionals (co-authors). Several changes have been adapted in the survey after validation. Inclusion Criteria: all participants who attended the monthly JC (pharmacy residents, interns, clinical pharmacists, pharmacists, and administrators) from 2010 to 2014 at King Abdulaziz Medical City, Jeddah, Saudi Arabia.

### 2.2. Administration of Survey

All participants were e-mailed the survey link in May 2014 explaining the objectives and were invited to respond to the survey. E-mail reminders were sent for non-responders on a regular basis.

### 2.3. Structure of the Survey

The survey comprised of several quantitative domains to assess the perception of learners/pharmacists towards the structured journal club including the introduction sessions presented at the beginning of each academic year, the selection of topics, the clinical pharmacist as a moderator, the layout of the journal club, the presenter’s interaction with the moderator, and the perception of the practicing pharmacists towards the journal club. Each domain consisted of several questions. The first author was the same JC moderator who consistently organized the JC from 2010 to 2014. Additionally, structures of the JC and appraisal forms have been consistent during the same period. The survey allowed participants to omit and skip different patterns for questions to direct respondents to answer questions relevant to their level of practice (pharmacy interns versus clinical pharmacists, for example)

### 2.4. Structure of the Journal Club

**Figure 1:**
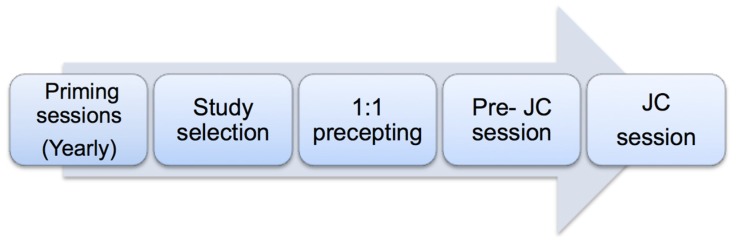
Structure of the journal club (JC).

We designed a structured journal club (Figure 1) since 2010 in the pharmaceutical care department accredited by the American Society of Health System Pharmacists (ASHP) for PGY1 residency. The aim of the journal club was to empower learners at different levels (interns, new practitioners, and residents) with basic principles of interpretation of literature via monthly sessions.

1. The structure of the JC starts with each academic year by introducing evidence-based medicine principles through priming sessions prepared and presented by clinical pharmacists. These are composed of three interactive sessions to address: (1) the main JC objectives, structure, and layout; (2) the basic concepts of study designs and measures of associations; and (3) tools to assess internal validity, interpretation of study results and generalizability. Moreover, a monthly schedule for a JC meeting is planned and distributed to clinical pharmacists and learners at the beginning of each academic year. Each academic year, we have a new batch of interns and residents who typically attend priming sessions. Interns attend 1–7 sessions of scheduled monthly JC based on their rotations at our site. However, our PGY1 residency program is for 2 years; therefore, most residents attend on average 18–20 sessions during their residency, and it varies for clinical pharmacists according to their schedule.
2. The selection of the study question whether it is a “practice-based clinical question” or a “new drug request for formulary addition” used to be decided by the clinical moderator; however, the JC presenting pharmacy intern or resident were required to conduct a literature review and identify potential studies that best address the question raised by the JC. Subsequently, the selection of the JC study occurs after discussion with the JC moderator. Finally, the clinical pharmacist JC moderator sends an e-mail notification of the selected study to pharmacy interns, residents and all pharmacy staff at least 1–2 weeks prior to the scheduled JC.
3. A one-to-one precepting followed to aid the presenter to best interpret the study findings using specific appraisal tools adapted from critical appraisal skills programs (CASPs), a Center of Evidence-Based Medicine appraisal, and other literature resources for assessment of multiple treatment comparisons [10,11,12]. In addition, several other checklists for trials reporting were discussed with the learners, depending on the study design presented in JC, such as a Consolidated Standards of Reporting Trials (CONSORT) statement for reporting randomized controlled trials, a Strengthening the Reporting of Observational studies in Epidemiology (STROBE) statement and a Preferred Reporting Items for Systematic Reviews and Meta-Analyses (PRISMA) statement for the reporting of meta-analysis [13,14,15].
4. Before starting the journal club, a 30–60 min separate interactive group “pre-JC session” is delivered to the interns and residents to discuss general epidemiological concepts of the paper or to highlight various aspects of the study design. This is usually followed by the journal club session (1 h), which is attended by pharmacy interns and residents, practicing pharmacists at different levels, and administrators.
5. The JC session starts with a small presentation of 15–20 min prepared by the learner (intern or resident) and includes the background, the study question in a PICOT format (patients/population, intervention, control, and time of the study), the main aspects of the study, and the critical appraisal of the topic. This was followed by an interactive group discussion moderated by the clinical pharmacist for critical appraisal of the study using the specific tools mentioned above. Sometimes the JC is presented again in a separate session to a multi-disciplinary team.

### 2.5. Sample Size Calculation

A sample of 86 participants were recruited to the study because we calculated that 86 respondents would provide 95% confidence intervals that would be sufficiently precise, to ±11%, even with a worst-case response of 50% [16].

### 2.6. Statistical Analysis

Responses are imported from SurveyMonkey [17] to Microsoft**^®^** Excel**^®^** for Mac 2011 version 14.7.0. Baseline demographics are presented as proportions; survey responses are also presented as proportions with 95% confidence intervals. Microsoft Excel^®^ for Mac 2011 version 14.7.0 and STATA 2014 (StataCorp LLC, College Station, TX, USA) were used for statistical analysis.

### 2.7. Ethics

The study has an Institutional Review Board consent waiver (IRBC/598/15) by King Abdullah International Medical Research Center.

## 3. Results

**Figure 2:**
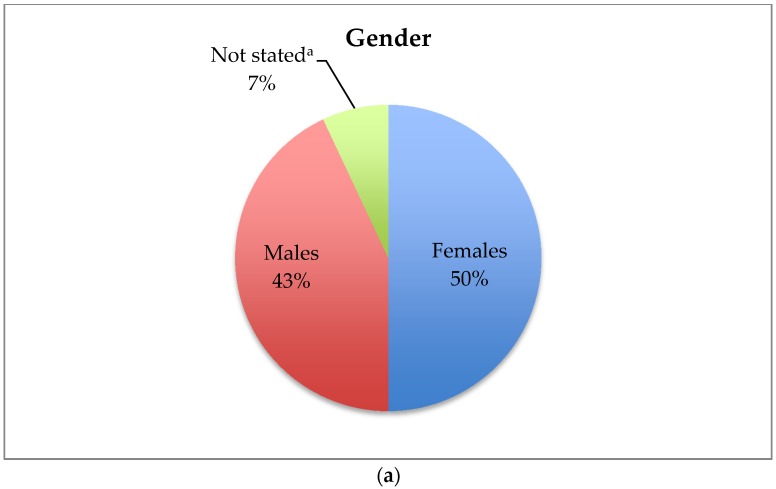

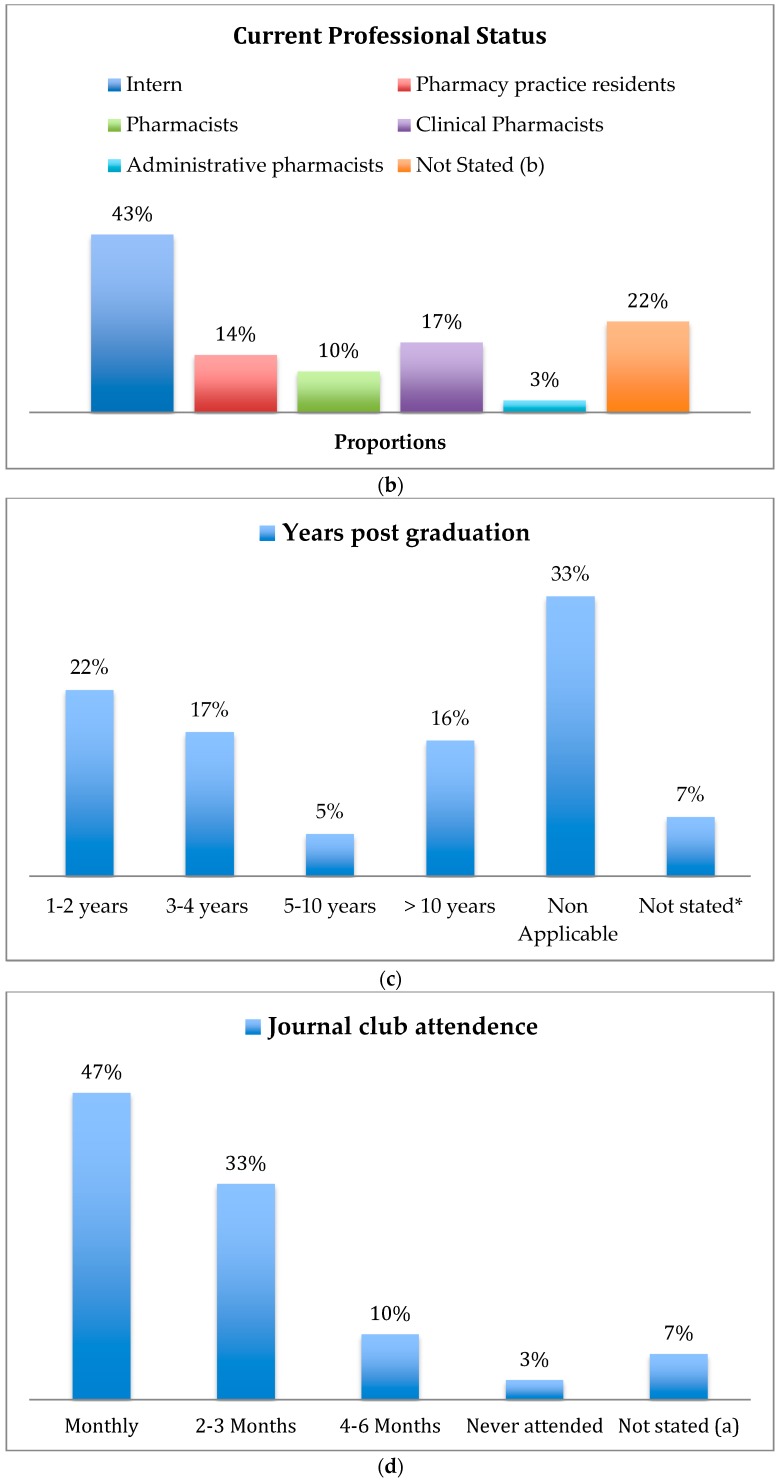

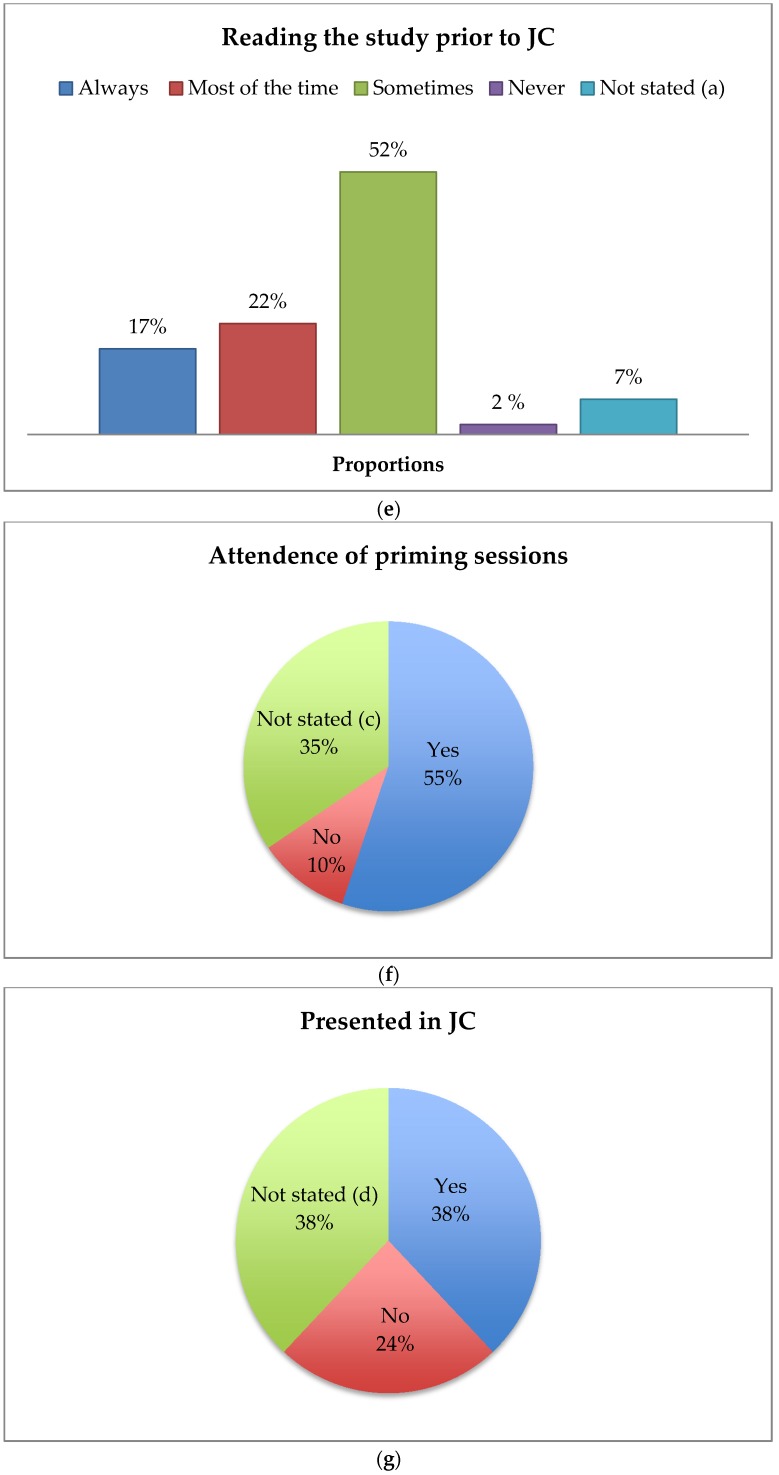

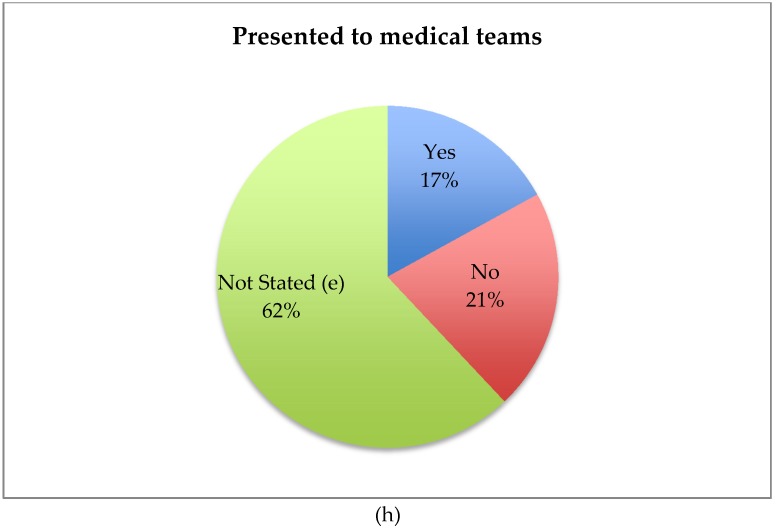
Baseline characteristics (a total of 58 participants): (**a**) gender: three refused to participate and one was not reported; (**b**) current professional status: three refused to participate and 10 were not applicable; (**c**) years post graduation: four were not stated and 19 were not applicable (pharmacy interns); (**d**) journal club attendance: three refused to participate and one was not applicable; (**e**) reading the study prior to JC; four were not stated (**f**) attendance of priming sessions: three refused to participate and 17 were not applicable (priming sessions are only directed to residents and interns); (**g**) presentation in JC: three refused to participate and 19 were not applicable as the question (presentation in the JC is mainly aiming for interns and residents (27/58); (**h**) presentation to medical team: three refused to participate and 33 were not applicable as the question for the presentation to medical teams is optional per the advice of the moderator.

The response rate was 58/89 (65%) of the invited participants, and only 3/58 (5%) refused to participate. Baseline characteristics of participants are presented in Figure 2.

Most of the response questions showed favorable positive responses for JCs among the collapsed response categories, except for one question related to materials for preparation of the journal club and the perception of clinical pharmacists vis-à-vis changes in clinical practice. Questions and response results are presented in Table 1.

## 4. Discussion

Our findings regarding our structured JC activity is consistent with various reports supporting the impact of JC on the perception of improving critical skills in interpretation of literature, and guiding decisions [2,3,5,18]. Additionally, its moderation by clinical pharmacist through imparting of specialized research training is unique in building competency in evidence informed decision-making. Priming sessions and 1:1 perceptorship were unique features in our JC with zero response for poor categories, which reflects the crucial role of clinical pharmacists in designing educational tools and mentoring.

Although 9/12 (75%) of clinical pharmacists believe that JC had been useful in changing clinical practice versus 3/12 (25%) disagreed. This might be explained by the small number of clinical pharmacists (12) in our survey and that many were not able to attend JC on a regular basis. 

There are several limitations to our survey for assessment of the structured JC: (1) We used the webpage service and emailed the survey, which may have limited the response rate to only 65% due to outdated email lists for participants in the past five years and undeliverable emails; (2) The survey was designed and validated to assess learner’s and pharmacist’s perception based on our structured JC, which might lead to subjective responses rather then pre- and post-test scores for an objective assessment of learner’s performance; (3) Our journal club did not host a biostatistician in most of our meetings, as is usually recommended for running effective journal clubs [8,19]; (4) The generalizability of our results for guiding formulary decisions and changing clinical practice shall be verified on a larger sample of practicing clinical pharmacists. 

However, our structured journal club has several strengths: (1) Our JC is mentored by a skilled clinical pharmacist, which is consistent with previous literature in improving critical appraisal skills and is tailored to individual learners by providing 1:1 learner-centered precepting [5,9]; (2) The Pre-JC Interactive session is unique to our structured JC to stimulate critical reasoning skills and introduces new epidemiological concepts to interns and residents; (3) Additionally, the discussion is fostered with the expertise of clinical pharmacists having diverse backgrounds and expertise, which creates a unique opportunity to learn and enrich the environment for empowering learners with critical thinking and decision-making for changing practice-based settings. Over the past five years, we have noticed a great improvement in the critical appraisal skills of learners attending the JC suggesting that our structured JC is a successful tool to guide pharmacy practitioners in various critical formulary and practice-changing decisions.

## 5. Conclusions

Our results suggest that clinical pharmacist steered journal club may serve as an effective tool to empower learners at different levels of pharmacy practice with evidence-based principles for interpretation of literature and guide informed decision-making.

Future studies with qualitative data, pre- and post-test scores, and an assessment of learning metrics shall provide robust evidence on the utility of a clinical pharmacist-steered JC in reshaping clinical practice and guiding formulary decisions.

## Figures and Tables

**Table 1:** The survey questions and the responses of the participants.

Questions	Responses	Proportions *n*/*N* (%)	95% Confidence Interval
**Overall perception of JC**			
1. Do you think JC activity adds to your knowledge and conceptual understanding of basic concepts during interpretation of the literature? ^a^	Yes	53/58 (89%)	0.81–0.97
2. Do you think the facilitation of JC is spoon-feeding and does not stimulate your critical thinking? ^a^	No	50/58 (86.2%)	0.77–0.95
3. Would you recommend JC activity to your colleagues to attend? ^a^	Yes	53/58 (91.4%)	0.84–0.99
4. What do you think about the competency of the clinical pharmacist moderating JC? ^b^	Poor ^c^	7/58 (12%)	0.036–0.20
	Neutral	3/58 (5.17%)	−0.005–0.11
	Good ^d^	43/58 (74%)	0.63–0.85
5. Do you think the clinical pharmacist steered JC idea is useful for you? ^b^	Yes	52/58 (89.6%)	0.82–0.97
**Introduction Sessions**			
1. How do you describe the introduction sessions for JC? ^e^	Poor ^c^	0/31 (0%)	-
	Neutral	2/31 (6.5%)	−0.02–0.15
	Good ^d^	29/31 (93.5%)	0.85–1.02
2. To what extent do you think the introduction session helped you to understand some basic concepts in critical appraisal skills? ^e^	Poor ^c^	0/31 (0%)	-
	Neutral	6/31 (19.4%)	0.055–0.33
	Good ^d^	25/31 (80.6%)	0.67–0.95
**Topic Selection**			
1. How far do you think the selection of the topics meet your core curriculum requirements for your internship/residency/board exams? ^f^	Rarely	7/36 (19.4%)	0.06–0.32
	Neutral	13/36 (36.1%)	0.20–0.52
	Always	16/36 (44.4%)	0.28–0.61
2. Do you think the topics discussed in the JC are current and help you to be updated with the literature?	Yes	35/36 (97.2%)	0.92–1.03
**Assessment of 1:1 Percepting**			
1. How do you evaluate your learning experience in preparation for JC? ^g^	Poor ^c^	0/22 (0%)	-
	Neutral	4/22 (18.2%)	0.021–0.34
	Good ^d^	18/22 (81.8%)	0.66–0.98
2. How do you describe your interaction with the JC Moderator during the preparation phase? ^g^	Poor ^c^	1 /22 (4.5%)	−0.04–0.13
	Neutral	5/22 (22.7%)	0.05-0.40
	Good ^d^	16/22 (72.7%)	0.54–0.91
3. How do you find the quality of the materials provided to you by the moderator to facilitate your understanding of the paper presented in the JC? ^g^	Poor ^c^	4/22 (18.2%)	0.02–0.34
	Neutral	3/22 (13.6%)	−0.01–0.28
	Good ^d^	15/22 (68.2)	0.49–0.88
**Formulary Decisions and Clinical Practice**			
1. Do you think JC activities facilitate formulary decisions? ^h^	Yes	10/12 (83.3%)	0.62–1.04
2. Do you think JC activities are useful in changing your clinical practice? ^h^	Yes ^i^	9/12 (75%)	0.51–0.99
	Neutral	2/12 (16.7%)	−0.04–0.38
	No ^j^	1/12 (8.3%)	−0.07–0.24

**^a^** Not stated: Three refused to participate and one was not applicable. ^b^ Not stated: Three refused to participate and two were not applicable. ^c^ Poor is a collapsed category for very poor and poor. ^d^ Good is a collapsed category of very good to excellent. ^e^ Twenty-seven not stated including 3 refused to participate and 24 were not applicable (not interns or residents). ^f^ Twenty-two not stated including 3 refused to participate and categories were collapsed to always for (mostly and almost always) and rarely for (sometimes and rarely). ^g^ Thirty-six not stated the questions including 3 refused to participate and 32 were not applicable (did not present). ^h^ Forty-six not stated including 3 refused to participate and 43 were not applicable. ^i^ Yes is a collapsed category for always and most of the times and ^j^ No is a collapsed category for not always and not at all.
